# The impact of family communication patterns on parent-child attachment and child quality of life

**DOI:** 10.1192/j.eurpsy.2024.502

**Published:** 2024-08-27

**Authors:** A. Z. Békefi, M. Miklósi, B. Szabó

**Affiliations:** ^1^Department of Developmental and Clinical Child Psychology, ELTE Eötvös Lóránd University Institute of Psychology; ^2^Department of Developmental and Clinical Child Psychology, ELTE Eötvös Lóránd University Institute of Psychology; ^3^Department of Clinical Psychology, Semmelweis University Faculty of Medicine; ^4^Autism Foundation; ^5^Heim Pál National Pediatric Institute, Centre of Mental Health; ^6^ELTE Eötvös Lóránd University, Doctoral School of Psychology; ^7^Developmental and Clinical Child Psychology, ELTE Eötvös Lóránd University Institute of Psychology, Budapest, Hungary

## Abstract

**Introduction:**

Previous research has demonstrated stable patterns of family communication. The Revised Family Communication Pattern Instrument (RFCP) is a common measure of these patterns. It posits two orientations: conformity orientation, characterized by a tendency to seek agreement within the family and by authoritarian decision-making; and conversation orientation, characterized by shared decision-making with the child and frequent family discussion.

**Objectives:**

The primary aim of our research was to adapt the RFCP questionnaire to the Hungarian language. Based on previous research, we hypothesized a negative relationship of conversation orientation and a positive relationship of conformity orientation with parents’ mentalizing problems, parental stress and burnout. According to our hypothesis, conformity orientation would predict both attachment anxiety and avoidance, whereas conversation orientation would decrease attachment anxiety.

**Methods:**

Parents of children aged 6–17 (*N*=269, female=86,2%, mean age=42,64 [*SD*=6,10] yrs) completed the following online questionnaires: Child Quality of Life Questionnaire (ILK) parent version, Reflective Function Questionnaire (RFQ-8), Experiences of Close Relationships Questionnaire (ECR-RS), Parent Burnout Questionnaire (PBA-HUN), Perceived Stress Questionnaire (PSS) and the RFCP instrument. We conducted a confirmatory factor analysis. Linear regression analyses predicting attachment anxiety and avoidance included two factors of the RFCP, the RFQ-8, the PBA-HUN, and the PSS total score, as predictors. In addition, two factors of the ECR-R were included in the linear regression analyses predicting quality of life.

**Results:**

The confirmatory factor analysis confirmed the original two-factor structure of RFCP (χ^2^=5482.21, *df*=325 *p*< .001, χ^2^/*df*=16.86, CFI=0.91, TLI=0.90, RMSEA=0.075 (90% CI 0.068–0.082)) and their internal reliability (Cronbach’s alpha = .78 and .74). Attachment avoidance (R^2^=0.12, *F*(5)=7.38, *p*< .001) was only predicted by conversation orientation (β=−0.28, *p*< .001), while attachment anxiety was predicted (R^2^=25.2, F(5)=17.7, *p*< .001) by conformity orientation (β=0.24, *p*< .001), parental mentalization difficulties (ß=0.20, *p*< .001) and parenting stress (β=0.15, *p*= .015). Parental report of the child’s quality of life was predicted most strongly by attachment anxiety (β=−0.28, *p*< .001), followed by conversation orientation (β=0.21, *p*< .001) and attachment avoidance (β=−0.18, *p*< .001, R^2^=28.8, *F*(7)=15.17, *p*< .001).

**Conclusions:**

The Hungarian version of the RFCP questionnaire has proven to be a reliable questionnaire. The importance of family communication patterns is demonstrated by the fact that it explains both the quality of parent-child attachment and the parent’s report on the child’s quality of life.

**Disclosure of Interest:**

None Declared

